# Clinical characteristics and establishment of a 2-year-OS predictive model of EGFR mutation-positive patients with pleural invasion of lung adenocarcinoma

**DOI:** 10.1097/MD.0000000000034184

**Published:** 2023-06-30

**Authors:** Qing Kong, Wei Wang, Qingqing Wang, Yuxia Yang, Gengye Chen, Tingshu Jiang

**Affiliations:** a Clinical Medical College, Weifang Medical University, Weifang, People’s Republic of China; b Yantai Yuhuangding Hospital, Yantai, People’s Republic of China; c Clinical Medical College, Yangzhou University, Yangzhou, China; d Respiratory Department of Emergency Center, People’s Hospital of Ningxia Hui Autonomous Region, Yinchuan, People’s Republic of China; e Department of Respiratory and Critical Care Medicine, Yantai Yuhuangding Hospital, Yantai, People’s Republic of China.

**Keywords:** 19, 21 L858R, 67, del, EGFR, Ki, lung adenocarcinoma, TKIs (epidermal growth factor receptor, tyrosine kinase inhibitors)

## Abstract

To investigate the differences between lung adenocarcinoma with the pleural invasion that has EGFR (epidermal growth factor receptor) 19-del or 21L858R mutations in terms of clinical characteristics and outcomes. EGFR mutation-positive patients with pleural metastasis of lung adenocarcinoma diagnosed in the Department of Respiratory Medicine of Yuhuangding Hospital of Yantai City, Shandong Province, from January 2014 to January 2022 were selected. The clinical data of the patients were collected to retrospectively analyze whether the clinical characteristics and prognosis of patients with 19-del or 21L858R mutation subtype were different and analyze the impact of clinical characteristics on the prognosis of patients. The difference in clinical characteristics between the 2 groups was analyzed by SPSS, *P* < .05. There was statistical significance. Univariate and multivariate regression analysis was performed with R soft. To establish a 2-year overall survival predictive model for patients with EGFR gene 19-del and 21L858R mutations in patients with pleural invasion of lung adenomas and to provide predictive model maps. Receiver operating characteristic curve, calibration curve, and decision curve analysis were used to evaluate the value of the prediction model in this study. Of the 74 patients included, the 19-del mutation group had a higher incidence of pleural thickening (*P* = .023) and a lower Ki-67 level (*P* = .035). There was no difference in 2-year overall survival and progression-free survival between the 2 mutations. There were differences in pleural thickening and Ki-67 index between the 2 groups, but no differences in disease outcome between the 2 groups. The nomogram model established based on gender, treatment regimen, CEA, lymph node metastasis, and pleural changes is accurate and feasible.

## 1. Introduction

Non-small cell lung cancer (NSCLC) poses a significant risk to people’s health due to its high morbidity and mortality rates. In NSCLC, lung adenocarcinoma is the most prevalent pathological form. In recent years,^[1]^ patients with advanced lung cancer now have much better survival owing to using epidermal growth factor receptor tyrosine kinase inhibitors (EGFR-TKIs),^[2,3]^ ushering in a new era of individualized therapy for lung adenocarcinoma. Currently, the clinical treatment of advanced lung adenocarcinoma is mainly based on molecular-targeted drugs. Treatment and prognosis assessment of lung adenocarcinoma benefit from early EGFR mutation diagnosis.^[4–6]^ For many patients, pleural effusion caused by pleural invasion is why they have already missed the chance for drastic surgery. EGFR-TKIs plays an essential role in the treatment of these patients. Forecast a patient’s prognosis and choose a treatment strategy in the early clinical stage. It is helpful to analyze the clinical traits and prognostic connection of patients with EGFR mutation.

A malignant tumor is a major public problem that seriously threatens human health. The incidence of lung cancer has risen to the second highest level in the world, and it is the tumor type with the most prevalence rate and fatality rate in China. This is the latest worldwide tumor data released by the International Institute for Oncology Analysis and Research in 2020. In 2020 alone, there were approximately 10 million cancer-related deaths worldwide and 19.3 million new cancer cases, with lung cancer accounting for 11.4% of all new tumor cases. One-eighth of all cancer patients who died were diagnosed with lung cancer.^[1]^ China alone is responsible for roughly half of all lung cancer fatalities worldwide.^[7]^ Their Pathological features and immunohistochemical examination show that lung cancer patients can be categorized into various types. Typically, patients can be separated into 2 groups, with non-small cell lung cancer accounting for around 2/5 of all lung cancer types. Non-small cell lung cancer includes squamous cell carcinoma, adenocarcinoma prone to gene mutation, and a small proportion of large cell carcinoma in smoking men. The incidence of lung cancer in men is not unambiguously greater than that in women when they are younger, according to clinical statistics, but it is significantly higher in men than in women after the age of 40 due to the fact that the majority of male patients are smokers. This can prove that perennial smoking is one of the causes of lung cancer; that is, perennial smokers are more likely to develop lung cancer.^[8]^ At the same time, among the causes of preventable lung cancer, not only smoking but also increasing haze, increasingly severe air pollution, occupational dust exposure, and other reasons have increased the incidence of lung cancer in varying degrees.^[9]^

The most recent Chinese Society of Clinical Oncology guidelines for treating non-small cell lung cancer state that individuals who meet the surgical criteria can have surgery and that patients with positive targeting genes should initially consider targeted therapy. Choosing appropriate radiotherapy, chemotherapy, and immunotherapy according to the different stages of lung tumors and patients’ general conditions is the primary treatment recommended by the current guidelines for lung tumors. For patients with early lung tumors, the latest Chinese Society of Clinical Oncology guidelines recommend grade I treatment as surgical treatment, which can be combined with local radiation therapy, but it still has defects, such as easy recurrence after operation.^[10]^ Nearly 80% of people with advanced lung cancer who receive a new diagnosis also have metastatic or locally advanced diseases. Once the disease develops to an advanced local stage or with metastatic tumor, the effect of surgery and radiotherapy is poor. The selection of appropriate chemotherapy, targeting, and immunotherapy drugs can achieve temporary palliative treatment, prolong the survival time, increase the likelihood of survival for 5 years, raise prospects, and lessen problems.

Adenocarcinoma is the most prevalent subtype of lung cancer, accounting for 50% or more of all diagnoses. Lung cancer incidence has increased in recent years.^[11]^ The prognosis of patients with lung adenocarcinoma is relatively poor, but studies have found that the 5-year survival rate of patients with clinical diseases with TNM (tumor-lymph node metastasis) stage IA is about 60%.^[12]^ Traditional diagnostic tools (including imaging, sputum cytology, tumor markers, etc.), due to lack of sensitivity and limited diagnostic conditions, 75% of patients are in the advanced stage of lung cancer at the time of diagnosis. Only 10% to 15% of new cases have been diagnosed in the early clinical stage despite ongoing advancements in diagnostic methods in recent years.^[13]^ To prevent metastasis, recurrence and lengthen longevity, it is crucial to assess the early prognosis of lung adenocarcinoma and provide each patient with a unique course of treatment.

As people realize that lung cancer does not develop suddenly but is the result of gradual changes in heredity and genetic epistemology, some progress has been made in studying lung adenocarcinoma-related genes, namely driving genes and tumor suppressor genes. A frequent driving gene in the development of lung cancer is EGFR gene mutation. After binding with growth factors, the transmembrane glycoprotein EGFR activates signal transduction pathways to control cell growth, differentiation, and proliferation. Those with early lung adenocarcinoma have the same rate of EGFR gene mutation as those with advanced lung cancer. Sequencing of the EGFR gene is equally essential in patients with early lung adenocarcinoma. There is little summary of the clinical characteristics of patients with early lung adenocarcinoma; only females, the elderly, and nonsmokers may be more likely to develop EGFR gene mutations than other early lung adenocarcinoma patients. The early detection and therapy of lung cancer called adenocarcinoma depend greatly on essential driving genes and tumor suppressor genes. For those with early-stage lung adenocarcinoma, according to their clinical characteristics, specific sequencing of the corresponding genes, combined with some micro biomolecule markers for comprehensive assessment, accurate risk assessment, and individualized treatment. The high mortality rate of lung cancer is mostly brought on by the lack of particular early-stage diagnostic tools and the disease’s propensity for spreading. Treatment of advanced NSCLC with EGFR-TKIs has many benefits, especially for those with the matching EGFR gene mutation. It can inhibit tumors by blocking the adenosine triphosphate binding site of cell receptors and blocking downstream signaling. EGFR gene mutations are complex and variable. All the mutations found so far are located in the tyrosine kinase region, mainly in exons 18-21. Different modifications of the EGFR gene have other effects on EGFR-TKIs. EGFR gene mutations were most common in exon 19 deletion mutation and exon 21 L858R missense mutation, accounting for 85% to 90% of the total mutations. Deleting exon 19 can change the Angle of the ATP-binding pocket, thus enhancing the sensitivity of tumor cells to small-molecule tyrosine kinase inhibitors. The L858R point mutation of exon 21 can improve A-loop stability and enhance tumor cells’ sensitivity to EGFR-TKIs. EGFR mutation is related to gender, ethnicity, smoking, and pathological type of patients, and the incidence of mutation is higher in the Oriental population, female, nonsmoking, and adenocarcinoma patients.

The metastasis of lung cancer to different organs is affected by various factors, and its treatment and prognosis are also entirely different. In clinical work, the influence of these clinicopathological features on lung cancer metastasis to other parts should be fully considered to formulate an appropriate treatment plan. Metastasis and recurrence are more frequent in patients with EGFR-positive mutations, and malignant pleural volume is more frequent in patients with EGFR-positive mutations. About half of NSCLC patients have advanced tumors at first diagnosis, and some have established distant metastasis. Malignant pleural effusion is a common complication of advanced NSCLC. The primary clinical manifestation of pleural invasion of lung adenocarcinoma, which is the cell type with the most frequent malignant pleural effusion and pleural effusion in patients with lung adenocarcinoma indicates that pleural tumor invasion has occurred, which suggests that the disease has entered the advanced stage or disease progression.

Targeted therapy has changed the treatment paradigm for NSCLC. As the standard adjuvant therapy for patients with stage II to IIIA NSCLC, adjuvant chemotherapy has reached a bottleneck recently, with limited patient benefits. With the discovery of novel medications and the development of gene identification technology, adjuvant targeted therapy is still being investigated, and the understanding and application of targeted therapy drugs are getting deeper and deeper. The mode of targeted therapy has gradually transitioned from the initial single-drug strategy to the multi-drug combination mode and has shown exciting promise and results. Targeted therapies may eventually change the lung cancer treatment paradigm, offering hope to patients with limited treatment options. At present, the mechanism of drug resistance of targeted drugs needs to be further explored, the best mode of targeted therapy still needs to be actively explored, and the search for the predictive factors of targeted drug response is still an important topic in clinical research.

To explore the differences in clinical features and outcomes between lung adenocarcinoma with EGFR19-del or 21L858R2 mutation and pleural invasion, we speculate that the above 2 groups of patients differ in some clinical features.

## 2. Methods

### 2.1. General data

Eighty-three patients with EGFR-mutation-positive lung adenocarcinoma were confirmed to have pleural invasion by thoracoscopic pleural biopsy in the respiratory Department of Yuhuangding Hospital, Yantai City, Shandong Province from January 2014 to January 2022 selected, and their clinical characteristics were collected. Medical thoracoscopy is performed by the experienced associate director or above physicians in Yantai Yuhuangding Hospital. Through the medical thoracoscopy, patients’ diseased pleura is directly observed and extracted for examination. All patients’ clinical data were obtained with their consent and signed informed consent.

Inclusion criteria and exclusion criteria: Inclusion criteria: Pleural histopathology of enrolled patients obtained under thoracoscopy indicated that the histopathological findings were consistent with the pathological findings of lung adenocarcinoma, which was consistent with the international clinical diagnostic criteria for lung adenocarcinoma.^[14]^ EGFR gene mutation was positive in pleural pathology; Complete medical records. Exclusion criteria: patients with one of the following conditions should be excluded: Patients with cognitive dysfunction and intellectual impairment. Incomplete clinical data. Patients with other primary tumors outside the lung; Pregnant and lactating women. All patients were newly diagnosed with lung adenocarcinoma and had not been treated with radiotherapy, chemotherapy, or surgical resection. EGFR gene mutation test negative patients. Patients with EGFR mutant subtypes other than 19-del and 21-L858R. Patients with favorable gene mutations other than EGFR; patients detected mutations in the 19-del and 21-L858R subtypes combined with other types of EGFR mutations.

### 2.2. Variables

Clinical characteristics of EGFR gene 19-del and 21L858R mutations were analyzed, including gender, age, smoking history, family history of tumor, location of pleural effusion, intrapulmonary metastasis, lymph node metastasis, distant metastasis, and thoracoscopic pleural manifestations. (Pleural lesions were divided into 5 types according to direct observation of pleura under medical thoracoscopy: Congestion, nodules, organisms, leucoplakia, thickening, and adhesion) and serum tumor markers. (Our hospital defined the positive limit of serum tumor indicators as SCCA (Squamous Cell Careinoma Antigen) ≥ 2.7 μg/L, CEA (carcinoembryonic antigen) ≥ 5 μg/L, CA199 (Carbohydrate antigen199) ≥ 39 U/L, NSE (neuron-specific enolase) ≥ 17 U/mL, CYFRA (Recombinant Cytokeratin Fragment Antigen 21-1) ≥ 3.3 ng/mL, CA125 (Carbohydrate antigen 125) ≥ 35 U/mL, CA724 (Carbohydrate antigen 724) ≥ 6.9 U/mL). Ki-67 index of pathological tissue (Ki-67 index was determined by the number of cells stained with Ki-67 antibody in tumor tissue and was represented by 11 grades: 0, 10, 20, 30, 40, 50, 60, 70, 80, 90, and 100%). The treatment options for patients were classified into 3 types: using the first generation of EGFR-TKIs, the third generation of EGFR-TKIs, and the third generation of EGFR-TKIs after the first generation of EGFR-TKIs resistance. The duration of disease progression and survival of patients were followed up.

### 2.3. Statistical analysis

The clinical aspects of the 19-del and 21L858R mutation groups, among which the ki-67 index was grade data in this investigation, were compared using the statistical program SPSS (25.0, CA). But this study applied the rank sum test to compare the variations in ki-67 expression levels between the 19-del and 21L858R groups. Using the χ^2^ test, which considers a *P* value of .05 to be statistically significant, the rates of clinical characteristics were compared between the 2 groups. In the predictive study, logistic univariate regression analysis was carried out using the R software (version R4.2.1) for gene mutation, clinical characteristics, treatment plans, etc. To screen the risk factors affecting the patients’ 2-year overall survival (OS) and progression-free survival (PFS). Multivariate logistic regression analysis was used to determine the 2-year risk factors for OS and PFS. In this paper, we developed a prediction of 2-year OS in patients with 19-del and 21 L858R pleural invasion. Receiver operating characteristic (ROC), calibration curve and decision curve analysis (DCA) were used to evaluate the value of the prediction model.

## 3. Results

### 3.1. EGFR gene mutation

Eighty-three patients with pleural invasion of lung adenocarcinoma with EGFR mutation-positive were screened, and 9 types of EGFR mutation were detected, including: 18 g719 × (1 case, 1.20%), 19-DEL (36 cases, 43.37%), 19 deletions combined with EML4-ALK (1 case, 1.20%), 20 exon insertion (1 case, 1.20%), 21 L858R (38 cases, 45.78%), 21 L858R combined with MET exon 14 (1 case, 1.20%), 21 L858R combined with 20 S768I (2 cases, 2.41%), 21 L858R combined with 20 T790M (1 case, 1.20%), 21 L861Q (2 cases, 2.41%).

### 3.2. Clinical features

Seventy-four patients with mutations in the 19-del and 21-L858R were statistically analyzed (Table 1). The incidence of pleural thickening in the 19-del group (13.9%) was higher than that in the 21L858R group (0%), and the difference was statistically significant (*P* = .023). There were no significant differences in gender, age, smoking history, pleural effusion site, pulmonary metastasis, lymph node metastasis, distant metastasis, and serum tumor markers (Table 2) between 19-del and 21-L858R mutation groups (*P* ≥ 0.05).

**Table 1:** Characteristics of 19-del and 21-L858R.

Characteristic		19-del	21L858R	*χ* ^2^	*P* value
Sex	Male	16 (44.4%)	14 (36.8%)	0.443	.506
	Female	20 (55.6%)	24 (63.2%)		
Age	≤50	5 (13.9%)	1 (2.6%)	1.815	.178
	>50	31 (86.1%)	37 (97.4%)		
Smoke	Negative	28 (77.8%)	31 (81.6%)	0.165	.684
	Positive	8 (22.2%)	7 (18.4%)		
Family history of tumors	Positive	13 (36.1%)	6 (15.8%)	1.001	.045
	Negative	23 (63.9%)	32 (84.2%)		
Pleural effusion	Left	16 (44.4%)	18 (37.4%)	0.064	.801
	Right	20 (55.6%)	20 (52.6%)		
Pleural manifestations					
Pleural congestion	Positive	31 (86.1%)	26 (68.4%)	3.269	.071
	Negative	5 (13.9%)	12 (31.6%)		
Pleural nodule	Positive	30 (83.3%)	29 (76.3%)	0.563	.453
	Negative	6 (16.7%)	9 (23.7%)		
Pleural neoplasms	Positive	3 (8.3%)	1 (2.6%)	0.325	.569
	Negative	33 (91.7%)	37 (97.4%)		
Pleural leukoplakia	Positive	9 (25.0%)	4 (10.5%)	2.674	.102
	Negative	27 (75%)	34 (89.5%)		
Pleural thickening	Positive	5 (13.9%)	0 (0%)		.023
	Negative	31 (86.1%)	38 (100%)		
Pleural adhesions	Positive	4 (11.1%)	3 (7.9%)	0.006	.94
	Negative	32 (88.9%)	35 (92.1)		
Intrapulmonary metastasis	Positive	4 (11.1%)	4 (10.5%)	0.000	1
	Negative	32 (88.9%)	34 (89.5%)		
Lymph node metastasis	Positive	8 (22.2%)	13 (34.2%)	1.307	.253
	Negative	28 (77.8%)	25 (65.8%)		
Distant metastasis	Positive	12 (33.3%)	11 (28.9%)	0.166	.684
	Negative	24 (66.7%)	27 (71.1%)		

**Table 2:** Serum tumor markers of 19-del and 21-L858R.

Serum tumor marker	Groups	Positive	*P* value
AFP (n = 62)	19-del (n = 30)	1 (3.33%)	.484
	21L858R (n = 32)	0	
CA125 (n = 63)	19-del (n = 30)	28 (93.3%)	.118
	21L858R (n = 33)	25 (75.8%)	
SCCA (n = 70)	19-del (n = 36)	1 (2.8%)	.00
	21L858R (n = 34)	0	
NSE (n = 71)	19-del (n = 36)	27 (75%)	.547
	21L858R (n = 35)	24 (68.6%)	
CYFRA (n = 71)	19-del (n = 36)	23 (63.9%)	.344
	21L858R (n = 35)	26 (74.3%)	
CEA (n = 71)	19-del (n = 36)	24 (66.7%)	.737
	21L858R (n = 35)	22 (62.9%)	
CA199 (n = 62)	19-del (n = 30)	11 (36.7%)	.652
	21L858R (n = 32)	10 (31.3%)	
CA72-4 (n = 62)	19-del (n = 30)	14 (46.7%)	.465
	21L858R (n = 32)	12 (37.5%)	

AFP = alpha fetoprotein, CA125 = carbohydrate antigen 125, CA199 = carbohydrate antigen 199, CA724 = carbohydrate antigen 724, CEA = carcinoembryonic antigen, CYFRA = recombinant cytokeratin fragment antigen, NSE = neuron-specific enolase, SCCA = squamous cell carcinoma antigen.

### 3.3. Ki-67 index in tumor tissue

The statistics of the ki-67 proliferation index of tumor tissue obtained by medical thoracoscopy in the 2 groups showed that the ki-67 index of 19-del was lower than that of the 21-L858R mutation (Table 3).

**Table 3:** Tumor tissue ki-67 index.

Mutation subtype	Quantity	M (P25, P75)	*Z*	*P* value
19-del	21	30% (10%, 55%)	−2.113	.035
21 L858R	26	40% (30%, 60%)	0.35	

### 3.4. Analysis of risk factors for 2-year OS and PFS

Logistic univariate regression analysis was performed on the clinical characteristics and treatment plans of 36 patients with 19-del mutation and 38 patients with 21-L858R mutation pleural metastasis, and the risk factors affecting 2-year OS and PFS were screened. The results showed that gender, lymph node metastasis, pleural leukoplakia, pleural congestion, and pleural adhesion had effects on OS in this study (*P* < .10), while gender, lymph node metastasis, pleural congestion, pleural leukoplakia, and pleural adhesion had effects on PFS (*P* < .10) (Table 4). The logistic multivariate regression study of the risk factors’ effects on OS and PFS included the risk factors from the logistic univariate regression analysis. Although there was no statistical significance in the multivariate analysis in the univariate analysis, gene mutation type, treatment regimen, and serum CEA level were included in the multivariate regression analysis considering the actual clinical situation. The results indicated that gender, lymph node metastasis, pleural hyperemia, pleural leukoma, pleural adhesion, treatment regimen, serum CEA level, and gene mutation type had no statistically significant effects on 2 years OS and PFS in patients with 19-del mutation and patients with 21-L858R mutant pleural metastasis (Table 5).

**Table 4:** Univariate analysis of the impact of clinical characteristics on 2-year OS and PFS.

Characteristic	2-year OS			PFS		
	Hazard. ratio	95% CI	*P* value	Hazard. ratio	95% CI	*P* value
Ki-67 index	1.13	0.26–4.9	.869	1.12	0.38–5.32	.889
Mutation subtype	1.15	0.6–2.18	.679	1.04	0.56–1.93	.909
Sex	0.56	0.3–1.06	.077	0.53	0.29–0.99	.045
Age	1.47	0.35–6.15	.595	1.18	0.36–3.88	.784
Smoke	1.52	0.73–3.13	.26	1.6	0.79–3.21	.188
Family history of tumors	1.43	0.65–3.12	.37	1.29	0.62–2.71	.498
Intrapulmonary metastasis	0.74	0.23–2.42	.615	0.72	0.22–2.35	.582
Lymph node metastasis	0.45	0.19–1.08	.074	0.49	0.22–1.11	.086
Distant metastasis	0.61	0.28–1.32	.21	0.58	0.27–1.27	.174
Pleural effusion	0.71	0.36–1.41	.331	0.67	0.34–1.32	.247
Pleural congestion	2.02	0.88–4.66	.099	2.13	0.93–4.87	.073
Pleural nodule	0.78	0.37–1.66	.523	0.82	0.39–1.74	.609
pleural neoplasms	0	0–Inf	.996	0	0–Inf	.996
Pleural leukoplakia	2.23	1.03–4.8	.041	2.48	1.18–5.21	.016
Pleural thickening	1.66	0.39–6.98	.492	2.49	0.75–8.23	.134
Pleural adhesions	2.34	0.95–5.75	.063	2.23	0.91–5.46	.079
CEA	1	1–1	.41	1	1–1	.385
AFP	0.87	0.66–1.16	.348	0.89	0.68–1.16	.391
CA199	1	1–1	.893	1	1–1	.86
NSE	0.99	0.96–1.02	.461	0.98	0.95–1.02	.348
CYFRA	0.99	0.92–1.06	.749	0.99	0.93–1.06	.834
CA125	1	1–1	.361	1	1–1	.343
CA72-4	1.02	0.98–1.05	.357	1.02	0.99–1.06	.132
Treatment 2	1.91	0.85–4.32	.119	2.13	0.95–4.75	.066
Treatment 3	1.72	0.79–3.75	.174	1.79	0.83–3.85	.137

Treatment plan 2 is the use of the third generations of targeted drugs, and treatment plan 3 is the use of the third generations of drugs after the resistance of the first generation of drugs.

AFP = alpha fetoprotein, CA125 = carbohydrate antigen 125, CA199 = carbohydrate antigen 199, CA724 = carbohydrate antigen 724, CEA = carcinoembryonic antigen, CYFRA = recombinant cytokeratin fragment antigen, NSE = neuron-specific enolase, PFS = progression-free survival, OS = overall survival.

**Table 5:** Multi-factor analysis of the influence of clinical features on OS and PFS in the past 2 years.

Characteristic	2-year OS			PFS		
	Hazard. ratio	95% CI	*P* value	Hazard. ratio	95% CI	*P* value
Sex	0.67	0.33–1.36	.269	0.63	0.31–1.27	.196
Treatment 2	2.18	0.87–5.43	.095	2.01	0.86–4.7	.107
Treatment 3	1.49	0.6–3.65	.388	1.39	0.61–3.21	.432
Mutation subtype	1.42	0.69–2.91	.339	1.42	0.69–2.91	.436
CEA	0.54	0.26–1.12	.097	1	1–1	.825
Lymph node metastasis	0.58	0.23–1.45	.245	0.55	0.24–1.27	.162
Pleural congestion	1.66	0.66–4.2	.285	1.97	0.78–5	.152
Pleural leukoplakia	2.37	0.84–6.63	.101	2.09	0.83–5.22	.116
Pleural adhesions	1.06	0.31–3.62	.921	0.98	0.3–3.16	.969

Treatment plan 2 is the use of the third generations of targeted drugs, and treatment plan 3 is the use of the third generations of drugs after the resistance of the first generation of drugs.

CEA = carcinoembryonic antigen, PFS = progression-free survival, OS = overall survival.

### 3.5. Prediction model

**Figure 1.**
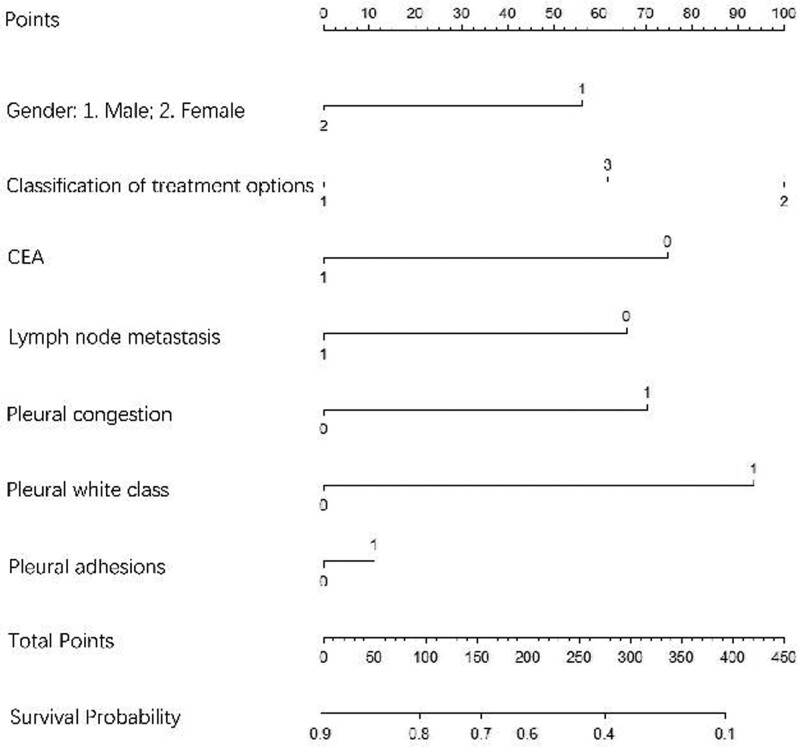
A nomogram model for 2-year survival. Treatment regimen1, 2 and 3 were: 1 generation of EGFR-TKI, 3 generations of EGFR-TKI, and 3 generations of EGFR-TKI after the progress of 1 generation of EGFR-TKI. The CEA is 1 in the elevated state and 0 in the normal state. Lymph node metastasis, pleural congestion, pleural leucoplakia and pleural adhesion were 1 positive and 0 negative. CEA = carcinoembryonic antigen, EGFR-TKIs = epidermal growth factor receptor tyrosine kinase inhibitors.

**Figure 2.**
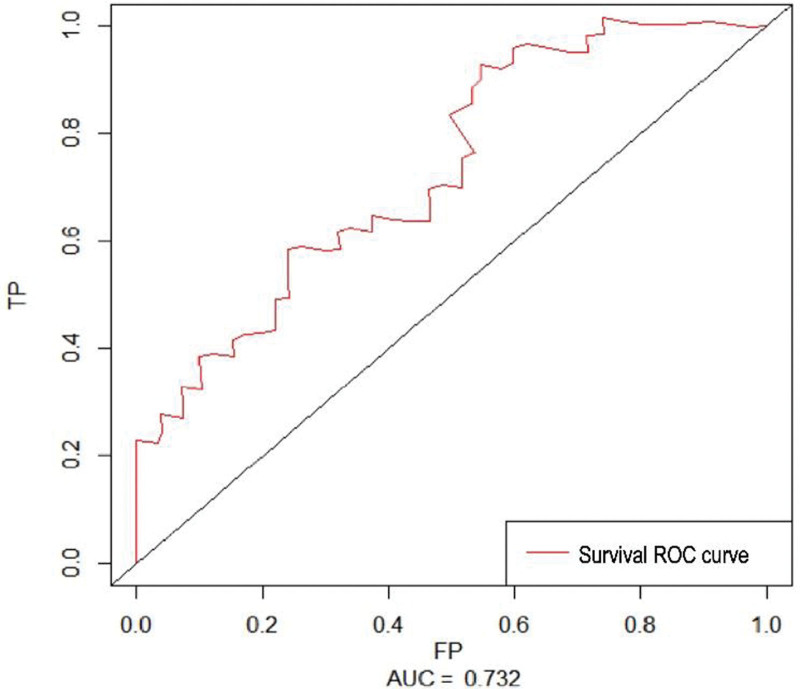
ROC curve. ROC = receiver operating characteristic curve.

Construction and evaluation of a 2-year OS prediction model. An understandable Nomogram prediction model was created using logistic regression analysis (Fig. 1). Patients’ gender, lymph node metastasis, pleural membranes, pleural congestion, pleural adhesion, treatment plan, and serum CEA level were used to predict 2-year OS in patients with pleural metastases with 19-del and 21L858R mutations. The 2-year OS of patients with 19-del mutation and patients with 21-L858R mutation pleural metastasis was taken as the state variable, the risk prediction made by line graph was taken as the test variable, and the clinical DCA curve of the nomograph model was drawn. The calculated area under the curve area under curve = 0.732. ROC curve and correction curve of the prediction model was drawn (Fig. 2). The prediction model of the prompt line chart has good accuracy.

**Figure 3.**
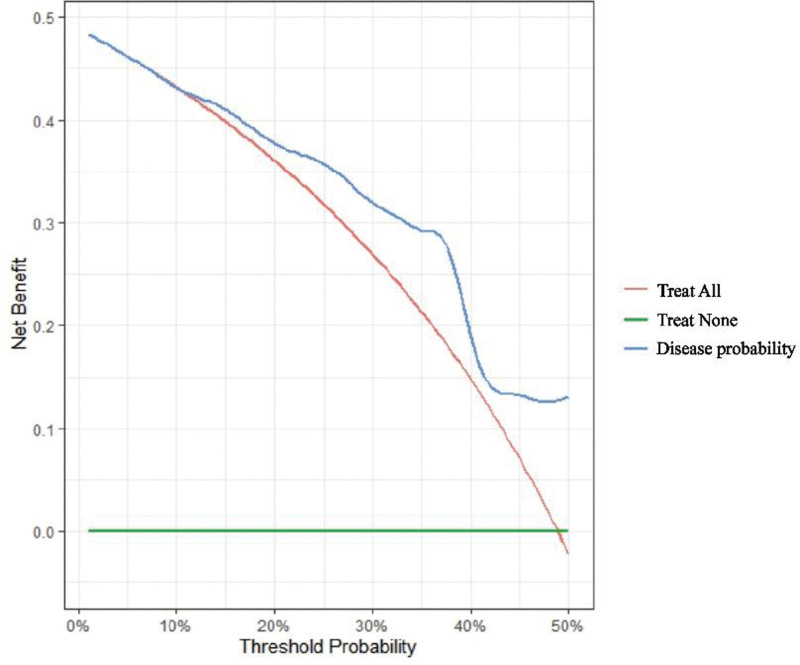
Decision curve analysis.

**Figure 4.**
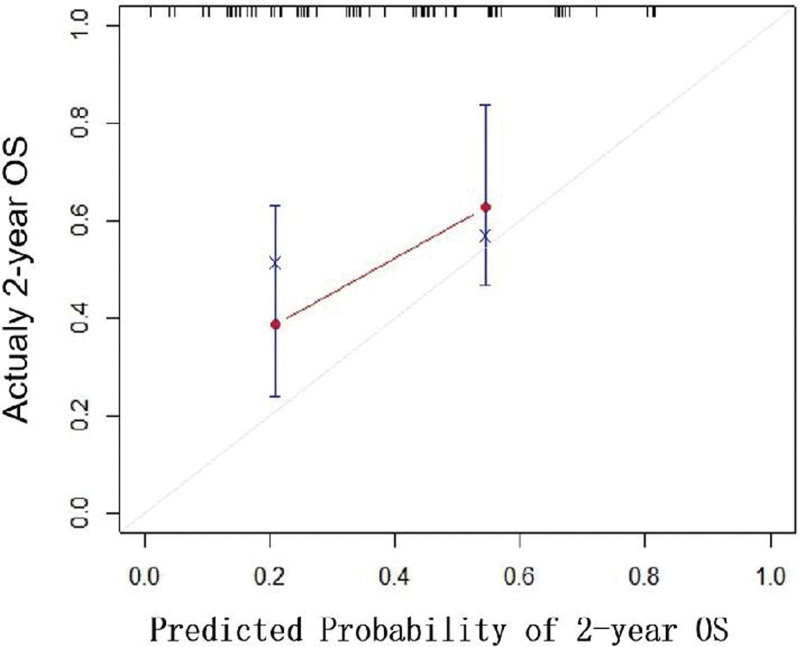
Correction curve.

The 2-year OS of patients with 19-del mutation and patients with 21-L858R mutation pleural metastasis was taken as the state variable, the risk prediction made by line graph was taken as the test variable, and the clinical DCA curve of line graph model was drawn (Fig. 2). The calculated area under the curve area under curve = 0. 732. The ROC curve and correction curve of the prediction model were drawn (Figs. 3 and 4). The prediction model of the prompt line chart has good accuracy.

## 4. Discussion

Lung cancer has some of the highest occurrence and mortality rates among all malignancies. The primary histological subtype of lung cancer is lung adenocarcinoma, and it is becoming more common each year.^[15]^ Although the application of computed tomography has realized the early diagnosis of lung cancer,^[11]^ there are still some patients with advanced stage at the initial diagnosis, and 15% of patients have pleural effusion at the initial diagnosis.^[16]^ Visceral pleural invasion (VPI) is currently one of the risk factors determining the prognosis of NSCLC, as shown by numerous research.^[17]^ VPI is currently one of the risk factors determining the prognosis of NSCLC, as shown by numerous research. The primary tumor of T1 size with VPI should be upgraded to T2a, and stage IA should become stage IB at the same time. However, the surgical resection technique and postoperative adjuvant chemotherapy will be directly influenced by tumor staging, impacting the patients’ survival. Consequently, preoperative VPI evaluation has significant clinical significance. For the prognosis of early NSCLC, VPI of lung cancer is a critical predictor of postoperative recurrence, pleural implantation, and bilateral lung metastases. EGFR19-del and 21L858R mutations increase the risk of VPI.^[18]^ EGFR-TKIs targeting^[19,20]^ is the recommended standard of care for patients with advanced EGFR-mutated NSCLC, and recommendations state that all NSCLC patients should have EGFR mutation testing performed at initial diagnosis.^[16]^ More than 200 types of EGFR mutations have been detected, among which there are differences in clinical characteristics, prognosis, and sensitivity to EGFR-TKIs among different mutation subtypes. One of the primary treatment targets for NSCLC is EGFR. However, EGFR gene mutations only exist in tumor tissues, particularly in NSCLC, and do not exist in healthy tissue cells. According to the current study, there is a relatively high rate of EGFR mutation and sensitivity to EGFR-TKIs therapy in nonsmoking, adenocarcinoma, and female patients. Somatic mutations of EGFR are mainly concentrated in exons 19 and 21. Only these 2 mutations can forecast how well EGFR inhibitors will work. Adjuvant EGFR-TKIs therapy for NSCLC patients can be guided by preoperative CEA level measurement, which may improve the total response rate of EGFR-TKIs. EGFR mutations mainly occur in exons 18 to 21, and conformational changes of different mutated channel proteins alter their activity and sensitivity to TKIs. Other mutations differ in lymph node metastasis and pathological subtypes. Mutations on chromosomes 18 to 21 are considered EGFR-TKIs sensitive, while modifications on 20-T790M are not sensitive to EGFR-TKIs.^[21]^ In around 1% of NSCLC patients, this mutation is hypothesized to include a threonine-methionine substitution in exon 20, boosting the mutant EGFR’s affinity to adenosine triphosphate competitively limiting the ability of reversible EGFR-TKIs to attach mutations on chromosomes 19 and 21 accounts for 90% of all modifications. Current studies generally believe that 19-del and 21-L858r are highly sensitive to EGFR-TKIs and can benefit from OS and PFS. In addition, compared with patients with 21-L858R, patients with 19-del who received TKIs had better overall survival, especially patients with advanced NSCLC.^[22]^ In conclusion, different mutation statuses of EGFR can be used as prognostic factors for patients.

In this study, patients with pleural invasion of lung adenoma were divided into the 19-del group and 21-L858R group. The relationship between clinical features and prognosis of the 2 groups was statistically analyzed. Different mutant subtypes of lung adenocarcinoma have different prognoses and treatment options. Similarly, the expression of other biomarkers also results in different prognoses. When comparing their clinical features, patients in the 21-L858R group were found to have a higher index of ki-67, a protein expressed only in proliferating cells. Both Ki-67 and EGFR mutations are involved in the proliferation and regulation of tumor cells, and both are independent factors affecting the prognosis of patients. The study of the relationship between ki-67 and EGFR is still controversial. The S, G2, and M phases of the cell cycle and portions of G related to proliferation all contain this protein, whereas G0 does not.^[23]^ It has been demonstrated that Ki-67 is necessary for cell growth^[24]^; it is created at the start of the cell proliferation cycle and is essentially destroyed at the end. Its actual purpose needs to be understood, though. The levels of Ki-67 protein were different in different cell proliferation cycles. Cells can be differentiated from static cells by immunohistochemical staining with a Ki-67 antibody.^[25]^ The percentage of proliferating cells in the tumor sample area is the tumor’s proliferation index, calculated by counting the number of cells in cancer stained with the Ki-67 antibody. The tissue slice’s Ki-67 index can be utilized to assess the tumor’s proliferative activity and contrast it with those of other tumors. Among gliomas, Shen-6 with a higher Ki-67 index showed more aggressive and invasive activity through glioma,^[26]^ bladder tumor,^[27]^ and anal carcinoma.^[28]^ As Scholzen and Gerdes reported in their review, the Ki-67 index predicted survival of multiple myeloma,^[25]^ soft tissue sarcoma, prostate cancer, and breast cancer. Demarchi LM and Reis MM found that in a long-term follow-up study of 64 patients with lung adenocarcinoma resection, Ki-67 was found to be an independent negative factor affecting the postoperative survival time of lung cancer patients. The same conclusion was also obtained in the studies of Hommura et al,^[29]^ Dosaka-Akita et al,^[30]^ and Giaccone et al.^[31]^ Both ki-67 and EGFR mutations are involved in the proliferation and regulation of tumor cells and are independent factors affecting the prognosis of patients. At present, the correlation between ki-67 and EGFR is still controversial. Several studies indicated that there was no correlation between the expression of ki-67 and EGFR mutation,^[32]^ but some studies also found that patients in the low expression group of Ki-67 were more prone to EGFR gene mutation than those in the high expression group of ki-67.^[33]^ Genetic analysis of lung adenocarcinoma pathways has shown that the cell cycle stimulation pathway is closely associated with prognosis and differentiation of lung adenocarcinoma subsets with increased solid components. In addition to being utilized for subclassification, the proliferation rate determined by measuring mitotic counts is also a known prognostic predictor for neuroendocrine lung cancers. As a proliferative marker, ki-67 has the potential further to subclassify NSCLC histological subgroups with varying prognostic outcomes. Ki-67’s predictive effects on various histological subtypes have yet to be widely supported by recent investigations. This study’s results showed a difference in the ki-67 proliferation index between lung adenocarcinoma 19-del and 21L858R genotype pleural infiltrating tumor, and the ki-67 level was lower in the 19-del group. In this study, the Ki-67 proliferation index was divided into a high-expression group (≥20%) and a low-expression group (<20%). In the 2-year univariate analysis of OS and PFS, differences in Ki-67 expression did not lead to differences in prognosis. In the multivariate analysis of 2-year survival and PFS, the difference in ki-67 expression between the 2 mutations did not lead to a difference in prediction, and the mechanism of this phenomenon needs further investigation.

A nomogram model is developed based on multivariate logistic regression analysis to forecast various scenarios accurately. Due to their capacity to condense a statistical prediction model to a single numerical estimate of the likelihood of an event, such as death or recurrence, specific to the circumstances of a single patient, nomograms have been frequently utilized for cancer prognosis. As an intuitive statistical expression, the Nomogram prediction model can simply and effectively quantify the risk.^[34,35]^ In this study, the logistic regression model results were used to build an intuitive and visual Nomogram prediction model (Fig. 1). Gender, lymph node metastasis, pleural leucoplakia, pleural congestion, pleural adhesion, treatment regimen, and serum CEA level of patients with pleural metastasis with 19-del and 21L858R mutations were included in the prediction model to predict 2-year OS. The chart can intuitively express the quantitative relationship between the relevant factors in the model. At the top of the graph, “Points” represents the score reference criteria for each predictor. The sum corresponds to “Total Points” at the bottom of the chart to obtain the final score. The predicted 2-year survival rate of this patient can be obtained by comparing it with the lower scale and “survival probability.” For example, a male patient with pleural metastasis of lung adenocarcinoma with elevated CEA, lymph node metastasis, and pleural adhesion observed under thoracoscopy was treated with first-generation EGFR-TKIs. The patient’s total score was 67, and the 2-year survival rate was 0.82. In the prediction model of this study, patients with male lymph node metastasis, low CEA level, pleural congestion, pleural leucoplakia, and pleural adhesion scored higher. The score of lymph node metastasis and high CEA level was lower. Patients treated with the first generation of targeted drugs alone scored more deficient than those treated with the first generation of targeted medicines resistant and then replaced with the third generation of targeted drugs. Patients who chose the third-generation drugs in the initial treatment scored the highest. High scores are associated with lower 2-year survival rates. In prediction research, the threshold with the maximum sum of sensitivity and specificity is usually selected as the optimal threshold. Still, this method is calculated based on the assumption that sensitivity and specificity are equally important. In practical decision-making, sometimes we are more concerned with the appearance of false positives or false negatives. Cost-benefit analysis in health economics can be a good choice for clinical forecasting models. Still, for forecasting models, this research method often difficult to achieve. Net income (NB) becomes an ideal alternative. The model was further explored through the decision analysis curve, and the results showed (Fig. 3) that determining whether patients should be treated with intervention based on the prediction model would improve clinical outcomes. The decision curve is above the Treat None and Treat All lines, so the model has clinical applicability and indicates that the prediction power of the model is high. The model’s calibration level was assessed using the findings of calibration curves, and a 1000-time bootstrap repeated sampling verification was carried out. Figure 4 shows the model’s calibration curve, where the gray line represents the idealized perfect agreement between model prediction and reality and the red line the model’s internal correction curve. Two straight lines are nearly parallel. The findings reveal that the projected probability of the model and the actual probability are in good agreement, demonstrating that the model’s ability to predict the 2-year survival rate is reliable.

In this study, we found that in patients with pleural invasion of EGFR-mutation-positive lung adenoma, patients with low ki-67 expression and a family history of the tumor had more 19-del mutations than those with the 21 L858R mutant subtype and patients with 19-del type were more likely to develop pleural thickened. This study showed no difference in prognosis between 19-del and 21 L858R mutation types. However, gender, lymph node metastasis, pleural leucoplast, pleural congestion, and pleural adhesion impacted 2-year OS and PFS in univariate analysis. However, in the multivariate analysis, the above clinical features, treatment regimen, and different mutation types did not affect the prognosis. The fundamental idea behind modern cancer treatment is that patients with specific cancer types and stages should receive care by a standardized, predefined regimen. The sensible use of targeted medicines can be aided by knowledge of the molecular alterations that occur in cancer. Patients with advanced lung adenocarcinoma with EGFR mutations benefit considerably from treatment with EGFR-TKIs. The usual first-line treatment for individuals with advanced EGFR-mutated NSCLC is targeted therapy with EGFR-TKIs. To effectively assess a patient’s prognosis at an early clinical stage and choose the most advantageous treatment option, it is necessary to analyze the association between various gene mutations, clinical characteristics, and prognosis.

## 5. Conclusion

This study found that in EGFR mutation-positive lung adenocarcinoma patients with pleural invasion, compared with 21-L858R mutation subtypes, patients with low ki-67 expression and family history of tumors were more likely to have 19-del mutations, and patients with 19-del type were more likely to develop pleural thickening. Among the patients with pleural invasion of EGFR mutation-positive lung adenocarcinoma, there was no difference in prognosis between 21-L858R and 19-del patients. The model established in this study to predict 2 year-OS in pleural leukoplakia, pleural hyperemia, pleural adhesion, treatment regimen, and serum CEA level in patients with EGFR mutation-positive lung adenocarcinoma pleural invasion in terms of gender, lymph node metastasis and gross pleural view under medical thoracoscope is accurate and feasible.

## Author contributions

**Conceptualization:** Qing Kong, Gengye Chen, Tingshu Jiang.

**Data curation:** Qing Kong, Wei Wang, Qingqing Wang, Yuxia Yang, Tingshu Jiang.

**Formal analysis:** Qing Kong, Wei Wang, Qingqing Wang, Tingshu Jiang.

**Investigation:** Qing Kong, Tingshu Jiang.

**Methodology:** Qing Kong, Tingshu Jiang.

**Project administration:** Qing Kong, Tingshu Jiang.

**Resources:** Qing Kong, Tingshu Jiang.

**Software:** Qing Kong.

**Supervision:** Qing Kong, Gengye Chen.

**Validation:** Qing Kong.

**Visualization:** Qing Kong.

**Writing – original draft:** Qing Kong.

**Writing – review & editing:** Qing Kong, Gengye Chen, Tingshu Jiang.
